# Biochemical Profiling and Physicochemical and Biological Valorization of Iraqi Honey: A Comprehensive Analysis

**DOI:** 10.3390/molecules29030671

**Published:** 2024-01-31

**Authors:** Omar Mohammed Hameed, Ohood Mzahim Shaker, Ahlem Ben Slima, Mohamed Makni

**Affiliations:** 1 Environmental Sciences and Sustainable Development Laboratory LASED, LR 18ES32, University of Sfax, Road of Aeroport Km 0.5 BP, Sfax 3029, Tunisia; omar.m.hameed@uosamarra.edu.iq; 2 Department of Pathological Analysis, College of Applied Sciences, University of Samarra, Samarra 34010, Iraq; uhood.m@uosamarra.edu.iq; 3 Department of Food Technology, High Institute of Biotechnology of Sfax, University of Sfax, Road of Aeroport Km 0.5 BP, Sfax 3029, Tunisia; ahlem.benslima@gmail.com

**Keywords:** honey, physicochemical properties, antioxidant, polyphenol, HPLC analysis, antimicrobial potency

## Abstract

Our study aimed to analyze five monovarietal honeys from the Salah Eddine region in Iraq, focusing on physicochemical, antioxidant, and antimicrobial properties and polyphenolic compounds. Our objective was to evaluate the strengths and qualities of Iraqi honeys, ensuring compliance with the Codex Alimentarius standard for honey. The spectrophotometric analysis included assessments of reduced sugar (75.8–77.7%), fructose-to-glucose ratio (0.7–0.9%), sucrose (2.2–2.9%), HMF (17.23–18.87 mg/kg), and melanoidin content (0.25–0.44), which were all determined. The electrical conductivity (0.39–0.46 mS/cm) using a conductivity meter, pH (4.02–4.31), and mineral composition were determined in all samples using atomic absorption spectrometry. Antioxidant activities were spectrophotometrically determined, through DPPH free radical scavenging (7.87–95.62 mg/mL), as was the total antioxidant activity (14.26–22.15 mg AAE/g), with correlations established with biochemical constituents such as the total phenol content, highlighting the significant presence of Coumaric acid (0.38–2.34 µg/mL), Catechin (1.80–2.68 µg/mL), and Quercetin (0.30 µg/mL) using HPLC. The study also observed notable antimicrobial activities using *Escherichia coli*, *Staphylococcus aureus*, and *Candida albicans* on Mueller–Hinton agar as well as through diffusion technique. In conclusion, our findings, including the antioxidant and antimicrobial strengths, underscore the substantial potential of Iraqi honeys in mitigating damage and preventing the onset of various diseases, affirming their good quality and adherence to international honey standards.

## 1. Introduction

Honey is a natural sweet material made by honeybees from floral nectar, which the bees collect and alter by mixing with specialized compounds of their own before depositing, dehydrating, storing, and leaving it in the honeycomb to ripen and develop [1]. 

Honey is primarily composed of natural sugars, including glucose and fructose, alongside small quantities of other carbohydrates, water, vitamins, minerals, proteins, and enzymes. The specific composition varies based on factors such as the floral source, geographical origin, and bee species. Functioning as a significant energy source with a high carbohydrate content (80–85%), honey’s sugars are easily digestible, akin to those found in many fruits [2]. While Bogdanov et al. [3] identified more than 22 sugars in honey, fructose and glucose emerge as the predominant. In nectar honey, the fructose content surpasses that of glucose [4]. Additionally, factors like the fructose/glucose ratio, the glucose/water ratio, and the overall sum of fructose and glucose are crucial in determining honey’s quality. The fructose/glucose ratio, for instance, indicates the honey’s ability to crystallize [2]. Its diverse flavors and aromas are intricately linked to the varied nectar sources [5]. The moisture content in bee honey plays a vital role in ensuring stability against fermentation and granulation. A low moisture content serves as a protective measure against microbial activity, extending the honey’s shelf life [6]. Furthermore, honey contains trace amounts of both enzymatic and non-enzymatic antioxidants, such as glucose oxidase, catalase, ascorbic acid, flavonoids, phenolic acids, carotenoid derivatives, organic acids, amino acids, proteins, and fragrant substances [7]. The concentration of these components is influenced by a range of factors, including the natural conditions during raw material collection, flower types, honeybee variety, and prevailing weather conditions [8].

Physicochemical analysis of honey plays a pivotal role in establishing the authenticity of the honey. Beyond its delicious taste and nutritional richness, the precise composition of honey is a key indicator of its origin and quality. Through comprehensive physicochemical analysis, including assessments of sugars, water content, acidity, and mineral composition, researchers can unveil the unique fingerprint of each honey variety, connecting it to specific floral and geographical sources [1]. This analytical approach is fundamental in discerning genuine, high-quality honey from adulterated or mislabeled products.

The antioxidant properties of honey, attributed to bioactive compounds such as flavonoids, phenolic acids, and enzymes, play a crucial role in neutralizing free radicals and preventing oxidative stress within the body [9]. Oxidative stress is linked to various chronic diseases, aging processes, and cellular damage. Assessing the antioxidant activities of honey provides insights into its potential to mitigate oxidative damage and reduce the risk factors that are associated with conditions like cardiovascular diseases, cancer, and neurodegenerative disorders [9]. Additionally, antioxidant-rich honey contributes to overall well-being and supports the body’s defense mechanisms against environmental stressors. The presence of important antioxidant bioactive molecules makes honey a versatile and valuable natural product, both in culinary and therapeutic applications. Researchers continue to explore its potential uses and health effects in various contexts [10,11,12]. Different types of honey may have varying effects due to their unique compositions, and the specific benefits may vary depending on the quality and source of the honey [13,14]. 

Honey’s well-documented antibacterial and antifungal activities make it a valuable natural agent in combating microbial infections. The presence of hydrogen peroxide, phenolic compounds, and other antimicrobial and antifungal constituents in honey contributes to its ability to inhibit the growth of bacteria and fungi [15,16]. Assessing the antibacterial and antifungal activities of honey is essential not only for its therapeutic applications in wound healing and infection management but also for ensuring its safety as a consumable product. While it is not a substitute for conventional medical treatment, some research suggests that it may have a role in managing or preventing parasitic infections. A study investigated the antiamoebic activity of honey in an in vitro model and demonstrated significant inhibitory effects against *Entamoeba histolytica*, the causative parasite of amoebiasis [16]. Research has explored the potential of honey to inhibit the growth of *Plasmodium falciparum*, the parasite that is responsible for malaria. It was also demonstrated that certain types of honey have antimalarial properties [17]. Honey has also been tested for its potential to inhibit the growth of *Toxoplasma gondii*, the parasite that is responsible for toxoplasmosis [18].

Iraqi honey holds a legendary status, with some considering it among the finest globally. The country’s diverse terrain, encompassing flora like date palms, citrus fruits, acacia trees, sidr, jujube, and wildflowers, contributes to the rich variety of honey sources. The honey in Iraq exhibits diverse flavors and aromas, owing to its derivation from different floral origins. Unfortunately, studies focusing on its quality and characterization are scarce, and its production is primarily limited to traditional beekeeping practices [19]. The main goals of this work were to determine the physicochemical composition, the antioxidant capacities and polyphenol composition, and the antibacterial and antifungal activities of samples of famous honeys from Iraq to understand their potential health benefits. 

## 2. Results and Discussion

### 2.1. Analysis of Sugars in Iraqi Honey Samples

The analysis of various sugar contents serves as a method to differentiate genuine honey from adulterated varieties. A key distinguishing feature of honey is the higher concentration of fructose compared to glucose. Typically, honey comprises around 40% fructose and 30% glucose, although these proportions can be influenced by factors such as the storage time and temperature. Sucrose is present in minimal amounts, ideally not exceeding 5%, except in honey that is sourced from specific plants [20]. 

The examination of reduced sugar in the five honey samples revealed levels that were within the range of 75.8% to 77.7%, as outlined in Table 1; all examined honey samples contained sugars like fructose and glucose within the standard test range. Among the samples, HO4 exhibited the highest reduced sugar content at 77.7%, while HO3 contained the lowest at 75.8%. Notably, the fructose content surpassed that of glucose in each sample, a typical characteristic of natural honey. Additionally, sucrose was identified in all samples, adhering to the permissible 5% mass ratio limit for unadulterated honey.

Based on the data presented in Table 1, there is no apparent indication that the five honey samples have been adulterated with cheaper sweeteners. The fructose/glucose ratio was also typical for honey. The higher the glucose content of honey is, the faster it crystallizes. The fructose-to-glucose ratio in honey should ideally be between 0.9 and 1.35. A fructose-to-glucose ratio that is lower than 0.8 promotes faster honey crystallization, whereas a ratio that is greater than 0.9 promotes slower crystallization [20,21].

The total content of reducing sugar cannot distinguish pure from adulterated honey samples. For this purpose, other quality measurements should be carried out (e.g., glucose, fructose, sucrose content, and fructose/glucose ratio). The sugar content of honey is mainly fructose (38% *w*/*v*), glucose (31%), and to a lesser amount, sucrose (1%). Fructose is the sugar that is responsible for the sweetness of honey, while the glucose content depends upon the source of nectar [20].

Our findings are consistent with the results obtained by Kamal et al. [22], which indicated that the values of reducing sugar in tested honey are between 62.5 and 77.2%. Contrary to our findings, the honey samples examined by Kamal et al. were fluid, as the fructose-to-glucose ratio was 1.14–1.34, and the sucrose level was 1.74–5.96%. These results demonstrate the lack of adulteration and honey collection at the ideal maturation time, because a high sucrose content may result from the addition of commercial sugar or may be attributed to the early collection of the honey.

### 2.2. Moisture Contents

The moisture content of honey is a critical factor influencing its quality, stability, and resistance to spoilage through yeast fermentation. Higher moisture levels increase the likelihood of fermentation during storage, while lower moisture levels (<20%) extend the shelf life of honey, aligning with the standards for many commercial varieties [1,23].

In this study, the moisture content of the honey samples was determined: HO1 at 14.13 ± 0.06 g/100 g, HO2 at 16.07 ± 0.06 g/100 g, HO3 at 15.13 ± 0.06 g/100 g, HO4 at 13.53 ± 0.06 g/100 g, and HO5 at 14.10 ± 0.10 g/100 g (Table 2).

Our findings align with the international quality recommendations for honey set by Codex Alimentations [1]. Significant differences in moisture content were observed among the honey types, particularly between HO2 (16.07 ± 0.06) and HO4 (13.53 ± 0.06) samples (*p* < 0.05). HO4 exhibited the lowest moisture content, followed by HO1 and HO5, with HO2 having the highest level (14.13 ± 0.06, 14.10 ± 0.10, and 16.07 ± 0.06, respectively). The moisture content of honey is a crucial determinant of its ability to resist fermentation and granulation during storage. However, this content is influenced by the temperature and relative humidity during honey production in specific geographic locations [24]. Honey generally possesses a low moisture content, contributing to its extended shelf life and resistance to microorganisms. The exact moisture content can vary based on factors such as the honey type, environmental conditions, and processing methods, typically ranging between 17 and 20% water content, as recorded for Pine honey (*Pinus halepensis* L.) from Germany and Clover honey (*Trifolium alexandrinum* L.) from Egypt, whose moisture contents were 19.10 and 19.30, respectively [25,26,27].

### 2.3. The pH of Honey Samples

The pH value is linked with the number of organic acids that are present in the honey. It can also be influenced by various other factors such as the presence of inorganic ions, as well as extraction and storage conditions, which affect the structure, stability, and shelf life of honey, as well as the fermentation process [23]. Generally, honey is mildly acidic, with an average pH of 3.9. This acidity is due to the minor acid content of honey, mainly amino acids and organic acids that are responsible for the characteristic taste of honey [20]. 

The acidity levels of five honey samples were measured. The results presented in Table 2 confirm that all samples were acidic. Furthermore, these values fell within the standard limit of a pH of 3.40–6.10 [1], ensuring the freshness of the honey. Among the samples, HO3 exhibited the highest acidity, with a pH of 4.02 ± 0.03, followed by HO4 (4.12 ± 0.01), HO1 (4.20 ± 0.01), and HO5 (4.23 ± 0.02), while HO2 displayed the lowest acidity at 4.31 ± 0.01. No significant differences in pH values were observed among the five honey types (*p* > 0.05).

The pH values of the examined honey samples closely resembled those reported in honeys from various regions, including India, Algeria, Brazil, Spain, and Turkey, which ranged between pH 3.49 and 4.70 [28]. The elevated acidity of honey is linked to the fermentation of carbohydrates into organic acids, contributing to two key characteristics of honey: its distinctive flavor and its microbiological stability [19].

Typically, honey maintains a relatively low and acidic pH range, generally between 3.2 and 4.5, although occasional variations may occur. The pH of honey is influenced by factors such as the floral source, processing techniques, and storage conditions [29].

### 2.4. Electrical Conductivity (EC) of Iraqi Honey Samples

The level of EC is an important indicator of the quality of honey. This parameter depends on the mineral content of honey. It is the most useful quality parameter for the classification of monofloral honey. Elevated EC values are generally associated with a higher mineral content, as minerals facilitate electrical conductivity. The specific EC value can vary based on honey type and geographical origin, influenced by factors such as the floral source, soil composition, and processing methods [30,31].

The HO2 honey samples exhibited the highest electrical conductivity (EC) at 0.46 ± 0.01 mS/cm. In contrast, HO1, HO4, HO5, and HO3 samples displayed lower EC values at 0.43 ± 0.03, 0.39 ± 0.01, 0.41 ± 0.01, and 0.43 ± 0.01 mS/cm, respectively (Table 2). EC serves as a reliable criterion for determining the botanical origin and is routinely employed in honey quality control. All analyzed honeys adhered to the standard limit that is recommended by Codex Alimentations (≤0.8 mS/cm) [1], indicating the high quality of Iraqi honey.

Results found by Ceylan et al. [32] for sunflower honey (0.27 mS/cm) and those of Conti et al. [33] for Italian honeys (0.63 ± 0.37 mS/cm) are similar to our findings, and all are less than 0.8 mS/cm as recommended.

### 2.5. Hydroxymethyl-furfuraldehyde Content (HMF)

Hydroxymethylfurfural (HMF) is a naturally occurring organic compound that can be found in various foods, including honey. HMF is formed when sugars, such as fructose and glucose, break down due to high temperatures and acidic conditions, and it is often used as an indicator of honey’s quality and freshness. High levels of HMF may indicate improper processing, prolonged storage, or exposure to elevated temperatures, which can degrade the quality of the honey [34].

The results for the five studied Iraqi honey samples are 17.67 ± 0.15 mg/kg for HO1, 17.23 ± 0.25 mg/kg for HO2, 18.13 ± 0.15 mg/kg for HO3, 18.87 ± 0.06 mg/kg for HO4, and 18.30 ± 0.10 mg/kg for HO5, respectively (Table 2). The acceptable limit for HMF in honey samples varies among countries, typically being higher in hot tropical regions. The maximum allowable limit is set at 40 mg/kg [34].

The levels of HMF within the samples were determined to be within the acceptable range, indicating their freshness and lack of exposure to heating. HMF is generated during the caramelization process, which involves the direct dehydration of sugars under acidic conditions. The concentration of HMF tends to rise with prolonged storage time [35]. Fresh, unheated honey typically contains minimal or non-detectable HMF, suggesting that honey with low HMF values remains largely unaltered. A study from Pakistan supported this notion, revealing elevated HMF levels in branded honey samples compared to their fresh counterparts, indicating an increase during storage [36]. Increased HMF levels are associated with prolonged storage at high temperatures or overheating of honey samples. The acceptance level of HMF in honey samples is different among countries, being higher in hot tropical countries [20].

In a prior investigation of Saudi honey, 3 out of 13 samples exhibited exceptionally high HMF levels, leading researchers to infer overheating or prolonged storage. However, the HMF levels in the remaining 10 samples were below the permissible limits. The notably low levels of HMF in these honey samples implied that they were either not heated or were freshly harvested [37].

### 2.6. Melanoidin Content in Iraqi Honey Samples

Melanoidins are compounds that are generated in the late stages of the Maillard reaction from reducing sugars, and as they are considered secondary antioxidants, formed during the food processing and preservation, this ability has been broadly studied [38]. 

The formation mechanism, composition, and structure of melanoidins have been extensively studied in both model systems and melanoidins derived from various food sources. In model systems, melanoidins were observed to form through either the polymerization of recurring units of furans or pyrroles, as indicated by Tressl et al. [39], or originated from the degradation products of sugars, leading to polymerization through aldol-type condensation and/or intact carbohydrates, as reported by Cämmerer and Kroh [40] and Cämmerer et al. [41]. Alternatively, melanoidins were found to result from protein cross-linking that is facilitated by low-molecular-weight colored compounds, as suggested by Hofman [42].

The melanoidin fractions of our honey samples were determined, and Table 2 summarizes the obtained melanoidin values of the honeys. The melanoidin fraction content differed across honeys of diverse plant origins, with HO1 and HO3 having the greatest level (0.44 ± 0.01, 0.43 ± 0.01, respectively), and HO5, HO4, and HO2 having 0.38 ± 0.01, 0.37 ± 0.01, and 0.25 ± 0.04, respectively. These samples were not unreasonably high in melanoidin, which confirms the high quality of Iraqi honey.

### 2.7. The Mineral Composition of Iraqi Honey Samples

One of the factors that are taken into consideration while evaluating the nutritional value of honey is its mineral content. This may be used as a biomarker for heavy metal contamination in honey’s surroundings and as a possible indication of the honey’s geographic origin. It also reveals any potential contamination during the honey processing. This analysis tells beekeepers and customers about the quality of the honey. Furthermore, the mineral content aids in estimating the environmental quality of different regions. The chemical analysis of major minerals and heavy metals found in Iraqi honey is presented in Table 3.

According to our findings, (K) was the most abundant mineral in Iraqi honey, with a wide range from 111.08 ± 0.01 mg/kg for HO2 to 867.08 ± 0.03 mg/kg for HO4 honey. (Na) was the second most prevalent mineral in Iraqi honey samples, with concentrations ranging from 140.68 ± 0.50 for HO4 to 822.24 ± 0.27 mg/kg for HO1. (Ca) and (Fe) were also present in significant quantities, with values ranging from 7.33 ± 0.06 mg/kg to 25.37 ± 0.06 mg/kg and 1.85 ± 0.001 mg/kg to 8.87 ± 0.003 mg/kg, respectively.

Furthermore, the trace minerals (Cu), (Zn), (Ni), and (Cd) were identified at low concentrations in the Iraqi honey samples, and they ranged from 0.86 ± 0.003 to 2.43 ± 0.01 mg/kg, 0 to 4.48 ± 0.003 mg/kg, 0 to 0.25 ± 0.01 mg/kg, and 0 to 0.01 ± 0.001 mg/kg, respectively. The heavy element (Pb) did not appear in any of the honey samples, indicating that all samples were of high quality and purity. In comparison with previous studies, the values found for (K), (Na), (Ca), and (Mg) in honey from the United Arab Emirates [43], Morocco [44], and Spain [45] were similar.

### 2.8. Antioxidant Activities and Bioactive Compounds

#### 2.8.1. Total Antioxidant Activities

Variations in antioxidant activity can be attributed to the diverse botanical sources of honey and the presence of various antioxidant compounds such as flavonoids, phenolic acids, and diterpenes, each exerting distinct antioxidative effects [46]. 

According to our results, as depicted in Table 4, HO1 showed the lowest antioxidant activity value (14.26 ± 0.03 mg AAE/g), while HO2 honey samples presented the highest values (22.15 ± 0.04 mg AAE/g).

Research conducted by Hodnick et al. [47] highlighted that flavonoids with a higher number of hydroxyl groups undergo faster oxidation. The fluctuations in antioxidant activity arise from structural modifications, variations in hydroxylation, and the methylation levels of the substances [48]. The phenolic components of honey, particularly flavonoids, contribute not only to its antibacterial properties but also establish it as a substantial source of antioxidants, thereby enhancing its potential therapeutic value [49]. Furthermore, the determination of the total phenolic contents and antioxidant activity in honeys serves as valuable indicators for assessing their quality.

El-Haskoury et al. [50] reported that antioxidant activity ranges between 35.03 and 60.94 mg AAE/g for Moroccan honey. Additionally, Vinson et al. [51] noted that certain phenolic compounds, acting as antioxidants, exhibit varying response rates under similar conditions. Beyond phenolic compounds, the presence of elements like vitamins C and E, along with carotenoids, can influence the overall antioxidant activity [51].

#### 2.8.2. DPPH (1,1-diphenyl-2-picrylhydrazyl) Assay

The antioxidant activity of the tested samples was conducted by DPPH assay, which is one of the most stable free radicals and is frequently used in the evaluation of radical scavengers in natural foods. The DPPH radical scavenging effect serves as an indicator of honey’s comprehensive hydrogen/electron donation activity, like other dietary foods. This assessment relies on measuring the antioxidant’s reducing ability in countering the DPPH radical [52]. The value of the DPPH in honey samples was determined and is given in Table 4. The Iraqi honey’s DPPH presented IC 50 values as follows: HO1, 18.59, HO2, 28.53, HO3, 17.78, HO4, 7.87, and HO5, 95.62. 

According to these results, HO5 showed the lowest DPPH value, while the HO4 honey samples presented the highest DPPH values. Estevinho et al. [53] reported that dark honeys had DPPH inhibition values that were greater than 70%, while light honeys had inhibition values that were lower than 40%. Furthermore, the work of Silici et al. [54,55] investigated DPPH inhibition in 50 honey samples, with almost half of those samples exhibiting DPPH inhibition levels of more than 50%. 

The DPPH activities of Iraqi honey samples were similar to those reported in previous studies on Serbian honeys (11.16–48.48 mg/mL), and also Turkish honeys, which ranged between 12.56 and 152.40 mg/mL [50].

#### 2.8.3. Total Phenolic Compounds in Iraqi Honey Samples

The functional properties of honey are related to the number of natural antioxidants from molecules collected by bees and floral nectars. The antioxidant effects of honey were attributed to the presence of bioactive compounds, such as phenolic acids, flavonoids, ascorbic acid, carotenoids, catalase, and peroxidase, as well as Maillard reaction products in the composition of honey [56]. The results presented in Table 4 show that all tested Iraqi honeys contain significantly high total phenolic contents, ranging from 120.33 ± 0.58 mg GAE/100 g in HO1 to 55.33 ± 0.25 mg GAE/100 g in HO5. The honey samples HO3 (90.80 ± 0.61 mg GAE/100 g), HO4 (80.60 ± 0.10 mg GAE/100 g), and HO2 (65.57 ± 0.12 mg GAE/100 g) presented lower total phenolic contents compared with the other honeys. Honey HO1, which contains the highest total phenolic contents, may possess the best functional antioxidant properties.

The obtained results align with findings from other researchers. For instance, Wilczyńska [57] reported the total phenolic content of Polish honeys, ranging from 175.7 (rape) to 1895.2 (heather) mg GAE kg^−1^. In another study, Mellen et al. [58] found the total phenolic content in multifloral Polish honey to be between 611 and 990 mg GAE kg^−1^. In contrast, Bertoncelj et al. [59] observed lower values for total phenolic compounds in Slovenian honeys compared to our study. Their results varied from 44.8 mg GAE kg^−1^ in acacia honey to higher values in lime, multifloral, forest, and honeydew (241.4 mg GAE kg^−1^).

### 2.9. Phenolic Compounds

Polyphenol components are suggested to contribute to honey’s antioxidant potential. A tight relationship between the free radical scavenging activity and phenolic component level has been identified. The polyphenol’s properties and amounts vary depending on the harvest season, environmental circumstances, plants, floral sources, and factors affecting it during storage. 

**Figure 1:**
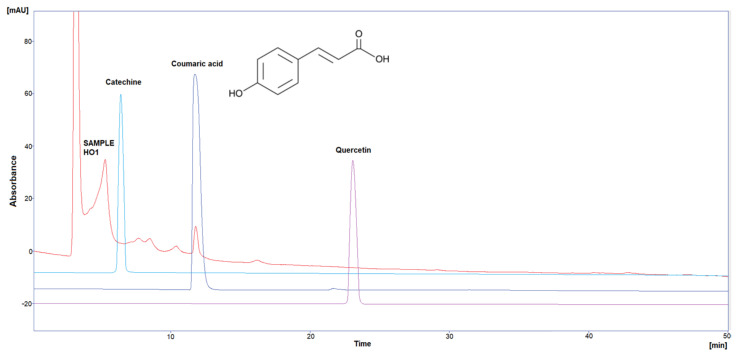
HPLC chromatogram of Catechin, Coumaric acid, and Quercetin in HO1.

**Figure 2:**
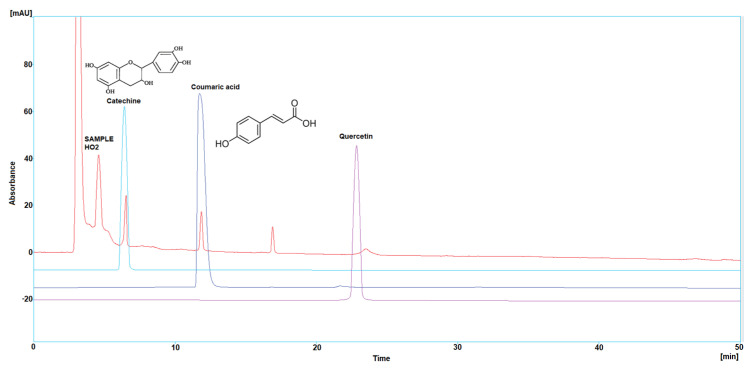
HPLC chromatogram of Catechin, Coumaric acid, and Quercetin in HO2.

**Figure 3:**
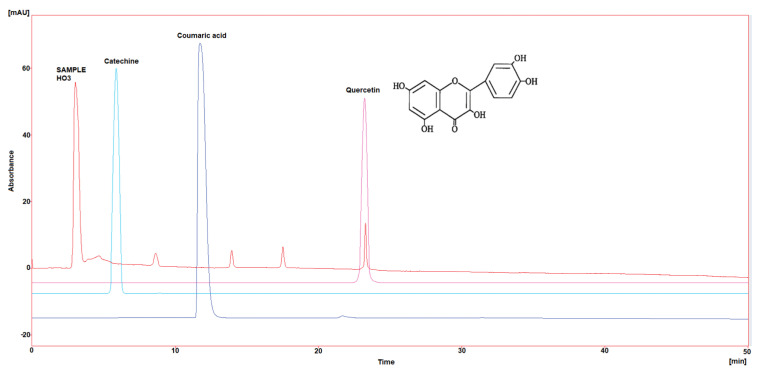
HPLC chromatogram of Catechin, Coumaric acid, and Quercetin in HO3.

**Figure 4:**
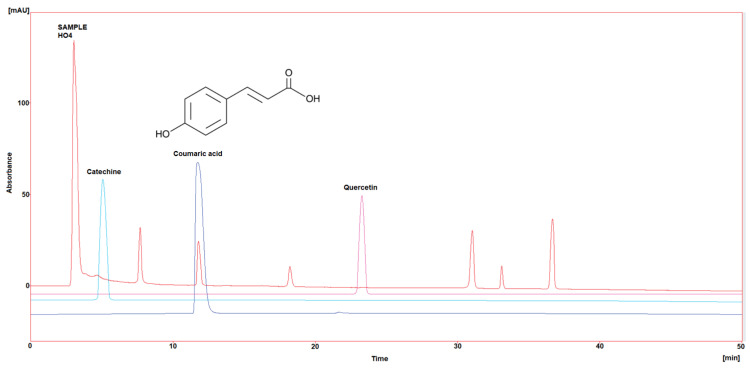
HPLC chromatogram of Catechin, Coumaric acid, and Quercetin in HO4.

**Figure 5:**
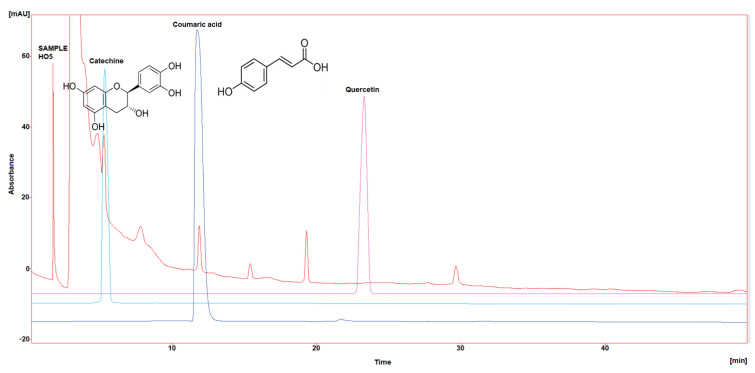
HPLC chromatogram of Catechin, Coumaric acid, and Quercetin in HO5.

Three polyphenol components were investigated, as they are largely identified in honey and specially in our Iraqi samples (Table 5 and Figure 1, Figure 2, Figure 3, Figure 4 and Figure 5). Coumaric acid presented the greatest value (2.34 µg/mL) in sample HO5, and the lowest value (0.04 µg/mL) in sample HO3. When compared to other samples of honey, HO3 displayed a higher Catechin level (2.68 µg/mL). Quercetin was found in high concentrations only in sample HO3 (0.3 µg/mL). 

There is evidence that honey contains roughly different types of phenolic compounds [60]. However, the profile of these phenolic compounds can vary depending on a variety of factors, including the floral source and the meteorological and geographical conditions. All honeys can contain quercetin, galangin, kaempferol, isorhamnetin, and luteolin. Quercetin is the most common flavonoid that is found in honey [61]. Most of the quercetin found in plants is linked to sugar moieties rather than being free [62]. These compounds are very important in preventing diseases and researchers confirm that in vivo, quercetin, catechin, and luteolin decrease blood platelet aggregation. Quercetin supplementation has been shown to have a significant capacity to reduce oxidative stress by scavenging reactive oxygen species (ROS), inhibiting xanthine oxidase, chelating metal ions, and lowering lipid peroxidation [63]. β-Coumaric acid is prevalent in honeybees’ natural diet and may act as a nutraceutical by influencing immunological and detoxification systems. β-Coumaric acid particularly increases the expression of all detoxification genes, as well as several antimicrobial peptide genes [64]. 

In a comparison between Iraqi and worldwide honeys, gallic and *p*-Coumaric acid are the dominant phenolic acids, as demonstrated also in Polish honeys [65], in Italian honey [66], and in Serbian honeys [67]. 

### 2.10. Antibacterial and Antifungal Activities of Iraqi Honey Samples

The well diffusion method results demonstrated robust antibacterial activity across a spectrum of bacteria and fungi for all tested honeys, inhibiting the growth of these microorganisms. The inhibition zone diameter for Gram-negative bacteria around honey-containing wells ranged from 9 to 14.4 mm, closely aligning with the diameter observed around antibiotic disks to which the bacteria were sensitive (Table 6). The inhibition zone is probably due to the original compositions of plant sources of honey such as *Eucalyptus grandis*, which is rich in bioactive molecules such as quinic, gallic, protocatechic, and ellagic acids and catechin [68,69]. Meanwhile, citrus phenolic compounds have been identified, and gallic, caffeic, sinapic, chlorogenic, β-coumaric, rosmarinic, trans-2-dihydrocinnamic, and cinnamic acids, as well as epigallocatechin, are identified [70]. 

Likewise, Gram-positive bacteria exhibited inhibition zone diameters ranging from 9.50 to 15.6 mm, which is consistent with antibiotic sensitivity. The fungi inhibition zones displayed diameters between 9.1 and 14.4 mm. Significant differences emerged among the three honey concentrations employed, with a 50% concentration proving most effective. These findings resonate with a local study by Jawad [71], demonstrating honey’s inhibition of Gram-positive bacteria, specifically *S. aureus*.

In line with these results, Mandal and Mandal [72] found inhibition zones around honey wells measuring approximately 13–14 mm for *E. coli* and 20–21 mm for *S. aureus*. Their study emphasized honey’s antibacterial efficacy against both Gram-positive and Gram-negative bacteria, particularly at concentrations of 40% or higher. Comparisons between honey and antibiotic antibacterial activities were explored by Osho and Bello [73], who observed honey’s peak effectiveness at 25% and 100% concentrations against various bacterial strains.

Taormina et al. [74] affirmed honey’s antibacterial activity against numerous pathogenic bacteria, noting that the floral source and honey concentration influenced the antibacterial effect. Al-Hasani [75] differentiated between red and white honey, finding superior antibacterial activity in red honey against clinical bacteria, including *S. aureus*. Hegazi et al. [76] and Shenoy et al. [77] also highlighted honey’s inhibitory effects on bacterial growth, influenced by both the pathogen type and honey concentration. Shenoy et al. further noted that higher honey concentrations were more effective, with shorter incubation durations.

Molan [78] emphasized significant variations in antibacterial activity results across studies due to differences in honey samples. The antibacterial capacity and bacterial response depend on various factors, notably the compounds that are present in honey, playing a crucial role in its antimicrobial activity [79,80].

## 3. Materials and Methods

### 3.1. Sample Collection

Five monovarietal honey samples were collected randomly from Salah Eddin market and from beekeepers during 2021 honey harvesting season (Table 7). All samples were stored at room temperature in a dark place until further analysis to avoid the effect of laboratory conditions on the chemical composition and physical/biological properties of honey samples. 

### 3.2. Reagent

The phenolic compounds, Coumaric acid, Catechin, and Quercetin were bought from Wuhan ChemFaces Biochemical Co., Ltd. (Wuhan, China), while the Folin–Ciocalteau reagent and anhydrous sodium carbonate, 2,2-diphenyl-1-picrylhydrazyl (DPPH), were from Sigma-Aldrich, Darmstadt, Germany, purchased from GAMA-tec company (Iraq-Babil, Iraq). The standards, including gallic acid and ascorpic acid, were purchased from Sigma-Aldrich (GAMA-tec company, Iraq-Babil, Iraq). All standard compounds have a purity of ≥95%. HPLC-grade acetonitrile, methanol, and formic acid were purchased from Sigma-Aldrich.

### 3.3. Sugar Analysis in Iraqi Honey Samples

#### 3.3.1. Total Sugar

For the quantification of total sugar, 50 µL of the sample was placed in a 96-well microplate. Subsequently, 150 µL of concentrated sulfuric acid was added, followed promptly by 30 µL of 5% phenol. The microplate was then heated for 5 min at 90 °C. After allowing it to cool to room temperature for an additional 5 min, the plate was wiped dry, and the absorbance at 490 nm was recorded. A glucose (50 mg/50 mL) served as the reference standard [81].

Total sugar (%) = (A sample)/(A standard) × Standard (%)(1)

where A sample = Sample absorbance, and A standard = Standard absorbance.

#### 3.3.2. Reducing Sugar and Concentration of Sucrose

The picric acid test is used to detect reducing sugars. First, 1 mL of the saturated picric acid was added to 1 mL of the test honey samples, followed by 0.5 mL of 10% sodium carbonate (10%) solution. Then, the test tube was heated in a boiling water bath and the absorbance was read at 490 nm [82].

Total Reduced sugar (%) = (A sample)/(A standard) × Standard (%)(2)

Sucrose (%) = Concentration of total Sugar − Reduced Sugar (%)(3)

where A sample = Sample absorbance, and A standard = Standard absorbance.

#### 3.3.3. Concentration of Glucose

The assessment of glucose relies on the enzymatic method using the RanDox kit [83]. The process involves enzymatic oxidation of glucose in the presence of glucose oxidase. The ensuing reaction between hydrogen peroxide and phenol, under peroxidase catalysis, produces a red-violet quinoneimine dye, serving as an indicator [84].

Glucose (%) = (A sample)/(A standard) × Standard Concentration (%)(4)

where A sample = Sample absorbance, and A standard = Standard absorbance.

#### 3.3.4. Concentration of Fructose

Fructose, a keto-hexose known as fruit sugar, is commonly found alongside sucrose in fruits like apples. Honey serves as a notable fructose-rich source. The determination of fructose concentration employed the method involving the formation of hydroxymethyl furfural from fructose in an acidic medium. In this approach, 2 mL of honey sample was combined with 1 mL of resorcinol reagent, followed by the addition of 7 mL of diluted hydrochloric acid. Simultaneously, 2 mL of the working standard, along with 1 mL of resorcinol reagent and 7 mL of dilute HCl, were pipetted out for comparison. A blank was included alongside the working standard. Subsequently, all tubes were heated in a water bath at 80 °C for precisely 10 min. After removal and cooling in tap water for 5 min, the color at 470 nm was recorded within 30 min [84].

Fructose (%) = (A sample)/(A standard) × Standard (%)(5)

Fructose to glucose ratio (%) = (Fructose)/(Glucose)(6)

where A sample = Sample absorbance, and A standard = Standard absorbance.

### 3.4. Determination of Moisture Content

The moisture content in the honey was determined through the utilization of the refractive index. A digital refractometer (NR 101 Spain) was employed, adjusted to 20 °C and calibrated using either distilled water or another certified reference material [85].

### 3.5. Determination of pH

A pH meter (HI 98127, Hanna instruments, Petite Riviere, Mauritius) was used to measure the pH of a 10% (*w*/*v*) solution of honey prepared in milli-Q water (Millipore Corporation, Billerica, MA, USA) [86].

### 3.6. Determination of Electrical Conductivity (EC)

The electrical conductivity (EC) was assessed with a HI 98311 conductivity meter from Hanna Instruments in Mauritius, employing a 20% (*w*/*v*) honey solution suspended in milli-Q water. The milli-Q water exhibited an electrical conductivity of less than 10 µS/cm [87].

### 3.7. Detection of Hydroxymethyl-furfuraldehyde (HMF)

The determination of HMF content followed the official method outlined by Sunkesula et al. [85]. Initially, 5 grams of each honey brand were liquefied in 25 mL of purified water. Subsequently, 0.5 mL of Carrez 1 and Carrez 2 solution (1:1 *v*/*v*) was introduced into the honey solution. The total volume was adjusted to 50 mL, using water in a volumetric flask, and the mixture was filtered. The initial 10 mL of the filtrate was discarded, and 5 mL of the honey solution was placed into two test tubes. In the first tube, 5 mL of purified water or honey sample was added, while in the second tube, 5 mL of a sodium bisulfite solution (NaHSO_3_ 0.2%) or a reference solution was introduced. The tubes were then transferred to 10 mL quartz cuvettes, and the absorbance was measured at 284 and 336 nm using a UV-visible spectrophotometer. The following equation was used to measure HMF:

HMF (mg/Kg) = (A284 − A336) × 149.7 × 5 × dilution factor/Sample weight (g)(7)

where A284 = absorbance at 284 nm, A336 = absorbance at 336 nm, 149.7, and 5 = constant theoretical value.

### 3.8. Determination of Mineral Elements

We added 5 mL of 0.1 M nitric acid to the honey, and the mixture was stirred on a heating plate until nearly dry. Subsequently, an additional 10 mL of the same acid was added, and the mixture was adjusted to a total volume of 25 mL with ultrapure water [50]. The mineral components were determined using atomic absorption spectrometry (Thermo Scientific ICE 3000 Series AA Spectrometer, Waltham, MA, USA). Prior to analysis, the instrument was calibrated with known concentrations of K (0.1–5 mg/L), Na (0.1–5 mg/L), Ca (0.1–5 mg/L), Fe (0.1–5 mg/L), Cu (0.5–4 mg/L), Zn (0.1–2 mg/L), Pb (0.1–4 mg/L), Cd (0.1–2 mg/L), and Ni (0.1–5 mg/L).

### 3.9. Determination of Melanoidin Content

Melanoidin content was estimated based on the Browning index by measuring the net absorbance of the honey samples [44]. After setting up the spectrophotometer according to the manufacturer’s instructions, the zero of the spectrophotometers using the blank cuvette filled with distilled water was adjusted. The absorbance of the sample at a specific wavelength (450 nm), known to be appropriate for melanoidin analysis, was measured as follows:

Total melanoidin (mg/mL) = (A sample)/(A standard) × Standard(8)

where A sample = Sample absorbance, and A standard = Standard absorbance.

### 3.10. Determination of Total Antioxidant Activity

The total antioxidant capacity of the honey samples was determined using the phosphomolybdenum technique with minor modifications, as reported by Mesbahi et al. [88]. In brief, 0.2 mL samples of honey were combined with a 2 mL reagent solution (0.6 M sulfuric acid, 28 mM sodium phosphate, and 4 mM ammonium molybdate). All tubes were sealed and incubated for 90 min in a boiling water bath at 95 °C. Using a UV visible spectrophotometer, the absorbance of the cooled mixture was measured at 695 nm against a blank sample. All tests were performed in triplicate, and the findings are presented as the mean average. 

### 3.11. DPPH (1,1-diphenyl-2-picrylhydrazyl) Assay

The electron-donating capacity of both samples and standards was investigated by assessing the bleaching effect on a purple-colored ethanol solution of 2,2-diphenyl-1-picrylhydrazyl (DPPH). In this spectrophotometric assay, the stable radical DPPH was employed as a reagent, prepared at a concentration of 0.002%. Various sample concentrations were placed in individual test tubes, and the volumes were adjusted to 2 mL using ethanol. Subsequently, 2 mL of DPPH solution (ranging from 2.0 to 0.001 mg/mL) was added to each test tube, and the solutions were left in the dark for thirty minutes. All samples were tested in triplicate, and the optical density was measured at 517 nm using a spectrophotometer. A control was established using ethanol with DPPH [89].

% Inhibition of DPPH activity = (A − B/A) × 100(9)

where A = Control absorbance, and B = Sample absorbance.

### 3.12. Determination of Total Phenolic Content (TPC)

The determination of total phenol in all samples was carried out using the Folin–Ciocalteau method [90] with slight modifications. Honey samples were dissolved in distilled water (50% *w*/*v*), and subsequently, 10 mL of the dissolved honey was combined with 180 mL of distilled water. This solution was then mixed with 10 mL of Folin–Ciocalteau reagent solution for 6 min, followed by the addition of 30 mL of 20% sodium carbonate solution to the mixture. After a 2 h incubation at room temperature, the absorbance was measured at 760 nm against a blank water solution. Gallic acid (GAE) served as the standard, and the total phenolic content was expressed as mg gallic acid equivalents per 100 g of honey (mg GAEeq/100 g). All tests were conducted in triplicate, and the results are presented as the mean average.

### 3.13. Determination of the Polyphenol Composition of Iraqi Honeys 

The polyphenol content of the honey samples was determined following the method outlined by Seal [91]. One mL of honey was combined with 5 mL of a 0.5 M NaOH solution and incubated at 65 °C with intermittent ultrasound for 12 h. The filtrate was collected post centrifugation (8000 rpm/10 min) and mixed with an equal volume of ethyl acetate using vortexing, followed by allowing the two layers to separate. The ethyl acetate layer was extracted, dried, and reconstituted with 2 mL of methanol for subsequent HPLC analysis (Knauer, GmbH, Berlin, Germany).

The HPLC analysis employed a C18 column (4.6 mm i.d., 5 µm particle size, 80 Å pore size). The mobile phase consisted of a 1% aqueous/acetic acid solution (Solvent A) and acetonitrile (Solvent B), with a flow rate set at 1 mL/min. The column temperature was thermostatically controlled at 28 °C, and the injection volume was maintained at 20 μL. A gradient elution was carried out by altering the proportion of solvent B to solvent A: from 10% to 40% B linearly over 28 min, from 40% to 60% B in 39 min, and from 60% to 90% B in 50 min. The mobile phase composition was then reverted to the initial condition (solvent B/solvent A, 10:90) over 50 min, followed by a 10 min run before injecting the next sample.

HPLC chromatograms were detected using a photodiode array UV detector at three different wavelengths (272, 280, and 310 nm). The detection of each compound was performed by matching retention time and absorbance spectrum of the standards (Catechin, Coumaric acid, and Quercetin), and the concentration was calculated by serial concentrations of external standard materials to build a calibration curve between concentration and its equivalent peak area.

### 3.14. Determination of the Antibacterial and Antifungal Activities of Iraqi Honeys

The honey’s antibacterial and antifungal activities were evaluated using the agar well diffusion technique [80]. The experiment was carried out as follows: First, 4–5 colonies of the tested isolated bacterial and fungi (*Escherichia coli*, *Staphylococcus aureus*, and *Candida albicans*) were taken from an overnight culture plate. The colonies were then emulsified in 5 mL of sterile normal saline until the turbidity was about similar to the McFarland No. 0.5 turbidity standard. After that, a sterile swab was dipped into the solution, and the bacterial or fungal isolate was inoculated on to the surface of a Mueller–Hinton agar plate (Himedia-India). Finally, three wells in the agar were cut with a sterile cork borer with a 6 mm diameter. Two wells were filled with 150 µL of honey solutions of 25%, 50%, and 75% (*v*/*v*), while the third well was filled with 150 µL of honey without any dilution (100%). We already used Penicillin G and Amphotericin-B as standard. The plates were incubated for 24 h at 37 °C. 

### 3.15. Statistical Analysis

Statistical analysis utilized analysis of variance (ANOVA), with all analyses conducted in triplicate. Statistically significant differences were determined for values with *p* < 0.05 at a 95% confidence interval. The results are presented as mean ± standard deviation.

## 4. Conclusions

Our study has provided new perspectives on the physicochemical characterization, polyphenolic content, and antioxidant activity of honey originating from the Salah Eddin/Iraq. The results consistently highlight the presence of abundant active biomolecules in these honeys, which is indicative of their high quality. This trait is mirrored by significant antioxidant and antiradical activities, observed consistently across all honey samples, irrespective of floral sources, geographic conditions, and environmental factors. The findings strongly imply that Iraqi honeys hold considerable potential in mitigating damage and preventing the onset of various diseases.

## Figures and Tables

**Table 1:** Sugar analysis of Iraqi honey samples.

	HO1	HO2	HO3	HO4	HO5	Average Standard Values
Reduced sugar before hydrolysis (%)	76.7	76.3	75.8	77.7	76.2	≥65
Fructose-to-glucose ratio (%)	0.9	0.9	0.7	0.9	0.8	0.9–1.35
Sucrose (%)	2.6	2.2	2.7	2.9	2.4	≤5

**Table 2:** Chemical properties of Iraqi honey samples.

Honey Sample	Moisture (g/100 g)	pH	EC (mS/cm)	HMF (mg/kg)	Melanoidin
HO1	14.13 ± 0.06	4.20 ± 0.01	0.43 ± 0.03	17.67 ± 0.15	0.44 ± 0.01
HO2	16.07 ± 0.06	4.31 ± 0.01	0.46 ± 0.01	17.23 ± 0.25	0.25 ± 0.04
HO3	15.13 ± 0.06	4.02 ± 0.03	0.43 ± 0.01	18.13 ± 0.15	0.43 ± 0.01
HO4	13.53 ± 0.06	4.12 ± 0.01	0.39 ± 0.01	18.87 ± 0.06	0.37 ± 0.01
HO5	14.10 ± 0.10	4.23 ± 0.02	0.41 ± 0.01	18.30 ± 0.10	0.38 ± 0.01

All results in the table show the mean of triplicates ± SD, *p* > 0.05.

**Table 3:** Mineral analysis of Iraqi honey samples.

Honey Sample	K (mg/kg)	Na (mg/kg)	Ca (mg/kg)	Fe (mg/kg)	Cu (mg/kg)	Zn (mg/kg)	Pb (mg/kg)	Ni (mg/kg)	Cd (mg/kg)
HO1	449.19 ± 0.29	822.24 ± 0.27	16.20 ± 0.10	1.85 ± 0.001	2.43 ± 0.01	4.48 ± 0.006	0	0.16 ± 0.001	0.002 ± 0.001
HO2	111.08 ± 0.01	268.36 ± 0.07	7.33 ± 0.06	2.64 ± 0.003	0.86 ± 0.003	0	0	0.022 ± 0.002	0
HO3	353.00 ± 0.10	187.72 ± 0.07	25.37 ± 0.06	8.87 ± 0.001	1.76 ± 0.01	0.34 ± 0.003	0	0	0
HO4	867.08 ± 0.03	140.68 ± 0.50	9.40 ± 0.10	6.10 ± 0.002	2.12 ± 0.002	0.18 ± 0.001	0	0	0.001 ± 0.001
HO5	243.87 ± 0.10	143.59 ± 0.01	9.67 ± 0.16	3.17 ± 0.001	1.16 ± 0.002	0.16 ± 0.004	0	0.25 ± 0.005	0.01 ± 0.001

All results in the table show the mean of triplicates ± SD, *p* > 0.05.

**Table 4:** Biochemical properties of Iraqi honey samples.

Honey Sample	Antioxidant Activity (mg AAE/g)	DPPH IC 50 (mg/mL)	Total Phenol (mg GAE/100 g)
HO1	14.26 ± 0.03	18.59	120.33 ± 0.58
HO2	22.15 ± 0.04	28.53	65.56 ± 0.12
HO3	15.25 ± 0.15	17.78	90.80 ± 0.61
HO4	16.17 ± 0.06	7.87	80.60 ± 0.10
HO5	17.29 ± 0.06	95.62	55.33 ± 0.25

All results in the table show the mean of triplicates ± SD, *p* > 0.05.

**Table 5:** Coumaric acid, Catechin, and Quercetin concentration in Iraqi honey samples.

Honey Sample	Coumaric Acid µg/mL Sample	Catechin µg/mL Sample	Quercetin µg/mL Sample
HO1	1.37	0.00	0.00
HO2	0.65	2.68	0.00
HO3	0.00	0.00	0.30
HO4	0.38	0.00	0.00
HO5	2.34	1.80	0.00

**Table 6:** Mean inhibition zone diameter (mm) of honeys on microbial strains.

Sample	HO1			HO2			HO3			HO4			HO5			Control (−)	Control (+)
	75%	50%	25%	75%	50%	25%	75%	50%	25%	75%	50%	25%	75%	50%	25%		
*Escherichia coli* Inhibition (mm)	10	12.4	9	11.2	12.6	11.1	12.2	14.4	9	12	13.6	11.2	11.7	12	11.5	0	12.6
*Staphylococcus aureus* Inhibition (mm)	11.7	12	13.2	9.5	12.1	11.2	12.2	15.6	11.3	13.2	14.5	10.1	12.5	14.3	11.2	0	13.1
*Candida albicans* Inhibition (mm)	9.3	12	11.6	13.1	14.4	12.6	13.5	16	12	13	14	12.5	9.1	13.8	12	0	14

**Table 7:** Iraqi honey sample collection.

Samples	Varietal Source	Location of Collection Salah Eddin (Iraq)
HO1	Citrus flower	Samarra
HO2	Clover	Tikrit
HO3	Thistle	Balad
HO4	Wild flower	Baiji
HO5	Eucalypus	Al Duloiya

## Data Availability

The data presented in this study are available on request from the corresponding author.

## References

[1] Codex Alimentarius (2001). Draft revised standard for standard for honey (at step 10 of the Codex procedure). Alinorm.

[2] White J.W., Doner L.W. (1980). Honey composition and properties. Beekeeping in the United States Agriculture Handbook Number 335.

[3] Bogdanov S., Ruoff K., Oddo L.P. (2004). Physico-chemical methods for the characterisation of unifloral honeys: A review. Apidologie.

[4] Aasima Z., Safdar M.N., Nouman S., Amer M., Tabassum H., Sial M.U. (2008). Chemical analysis and sensory evaluation of branded honey collected from Islamabad and Rawalpindi market. Pak. J. Agric. Res..

[5] Ouchemoukh S., Louaileche H., Schweitzer P. (2007). Physicochemical characteristics and pollen spectrum of some Algerian honeys. Food Control.

[6] Almasaudi S. (2021). The antibacterial activities of honey. Saudi J. Biol. Sci..

[7] Lachman J., Orsák M., Hejtmánková A., Kovářová E. (2010). Evaluation of antioxidant activity and total phenolics of selected Czech honeys. LWT-Food Sci. Technol..

[8] da Silva P.M., Gauche C., Gonzaga L.V., Costa A.C.O., Fett R. (2016). Honey: Chemical composition, stability and authenticity. Food Chem..

[9] Liu J.-R., Ye Y.-L., Lin T.-Y., Wang Y.-W., Peng C.-C. (2013). Effect of floral sources on the antioxidant, antimicrobial, and anti-inflammatory activities of honeys in Taiwan. Food Chem..

[10] Jull A.B., Cullum N., Dumville J.C., Westby M.J., Deshpande S., Walker N. (2015). Honey as a topical treatment for wounds. Cochrane Database Syst. Rev..

[11] Cohen H.A., Rozen J., Kristal H., Laks Y., Berkovitch M., Uziel Y., Kozer E., Pomeranz A., Efrat H. (2012). Effect of Honey on Nocturnal Cough and Sleep Quality: A Double-blind, Randomized, Placebo-Controlled Study. Pediatrics.

[12] Mahmood T., Akhtar N., Khan B.A., Naeem M. (2018). Exploring the potential of natural honey for preventing radiation-induced side effects in the oesophagus. CMB.

[13] Alvarez-Suarez J.M., Gasparrini M., Forbes-Hernández T.Y., Mazzoni L., Giampieri F. (2014). The Composition and Biological Activity of Honey: A Focus on Manuka Honey. Foods.

[14] Asha’ari Z.A., Ahmad M.Z., Din W.S.J.W., Hussin C.M.C., Leman I. (2013). Ingestion of honey improves the symptoms of allergic rhinitis: Evidence from a randomized placebo-controlled trial in the East Coast of Peninsular Malaysia. ASM.

[15] Carter D.A., Blair S.E., Cokcetin N.N., Bouzo D., Brooks P., Schothauer R., Harry E.J. (2016). Therapeutic Manuka Honey: No Longer So Alternative. Front. Microbiol..

[16] Mohammed S.E.A., Kabbashi A.S., Koko W.S., Ansari M.J., Adgaba N., Al-Ghamdi A. (2019). In vitro activity of some natural honeys against *Entamoeba histolytica* and *Giardia lamblia* trophozoites. Saudi J. Biol. Sci..

[17] Laksemi D.A., Tunas I.K., Damayanti P.A., Sudarmaja I., Widyadharma I.P.E., Wiryanthini I.A., Linawati N.M. (2023). Evaluation of Antimalarial Activity of Combination Extract of *Citrus aurantifolia* and Honey against *Plasmodium berghei*-İnfected Mice. Trop. J. Nat. Prod. Res..

[18] El Naggar H.M., Anwar M.M., Khayyal A.E., Abdelhameed R.M., Barakat A.M., Sadek S.A.S., Elashkar A.M. (2023). Application of honeybee venom loaded nanoparticles for the treatment of chronic toxoplasmosis: Parasitological, histopathological, and immunohistochemical studies. J. Parasit. Dis..

[19] Bogdanov S., Jurendic T., Sieber R., Gallmann P. (2008). Honey for Nutrition and Health: A Review. J. Am. Coll. Nutr..

[20] Aljohar H.I., Maher H.M., Albaqami J., Al-Mehaizie M., Orfali R., Alrubia S. (2018). Physical and chemical screening of honey samples available in the Saudi market: An important aspect in the authentication process and quality assessment. Saudi Pharm. J..

[21] Draiaia R., Dainese N., Borin A., Manzinello C., Gallina A., Mutinelli F. (2015). Physicochemical parameters and antibiotics residuals in Algerian honey. Afr. J. Biotechnol..

[22] Kamal M., Rashid H.U., Mondal S.C., El Taj H.F., Jung C. (2019). Physicochemical and microbiological characteristics of honey obtained through sugar feeding of bees. J. Food Sci. Technol..

[23] Terrab A., González A.G., Díez M.J., Heredia F.J. (2003). Mineral content and electrical conductivity of the honeys produced in Northwest Morocco and their contribution to the characterisation of unifloral honeys. J. Sci. Food Agric..

[24] El Sohaimy S.A., Masry S.H.D., Shehata M.G. (2015). Physicochemical characteristics of honey from different origins. Ann. Agric. Sci..

[25] de Sousa J.M.B., de Souza E.L., Marques G., de Toledo Benassi M., Gullón B., Pintado M.M., Magnani M. (2016). Sugar profile, physicochemical and sensory aspects of monofloral honeys produced by different stingless bee species in Brazilian semi-arid region. LWT-Food Sci. Technol..

[26] Smetanska I., Alharthi S.S., Selim K.A. (2021). Physicochemical, antioxidant capacity and color analysis of six honeys from different origin. J. King Saud. Univ.—Sci..

[27] Nurdin A.S., Saelan E., Nurdin I.N., Dustan (2021). Composition and nutritional content of Honey *Trigona* sp. in the Tikep forest management unit (KPH) North Moluccas. IOP Conf. Ser. Earth Environ. Sci..

[28] Saxena S., Gautam S., Sharma A. (2010). Physical, biochemical and antioxidant properties of some Indian honeys. Food Chem..

[29] Machado De-Melo A.A., Almeida-Muradian L.B.D., Sancho M.T., Pascual-Maté A. (2018). Composition and properties of *Apis mellifera* honey: A review. J. Apic. Res..

[30] Scripcă L.A., Amariei S. (2021). The Influence of Chemical Contaminants on the Physicochemical Properties of Unifloral and Multifloral Honey. Foods.

[31] Alqarni A.S., Owayss A.A., Mahmoud A.A., Hannan M.A. (2014). Mineral content and physical properties of local and imported honeys in Saudi Arabia. J. Saudi Chem. Soc..

[32] Ceylan D.A., Uslu N., Gül A., Özcan M.M., Özcan M.M. (2019). Effect of honey types on physico-chemical properties, electrical conductivity and mineral contents of honeys. J. Agroaliment. Process. Technol..

[33] Conti M.E., Canepari S., Finoia M.G., Mele G., Astolfi M.L. (2018). Characterization of Italian multifloral honeys on the basis of their mineral content and some typical quality parameters. J. Food Compos. Anal..

[34] Godoy C.A., Valderrama P., Boroski M. (2022). HMF Monitoring: Storage Condition and Honey Quality. Food Anal. Methods.

[35] Capuano E., Fogliano V. (2011). Acrylamide and 5-hydroxymethylfurfural (HMF): A review on metabolism, toxicity, occurrence in food and mitigation strategies. LWT Food Sci. Technol..

[36] Sajid M., Yamin M., Asad F., Yaqub S., Ahmad S., Mubarik M.A.M.S., Ahmad B., Ahmad W., Qamer S. (2020). Comparative study of physio-chemical analysis of fresh and branded honeys from Pakistan. Saudi J. Biol. Sci..

[37] Alqarni A.S., Owayss A.A., Mahmoud A.A. (2016). Physicochemical characteristics, total phenols and pigments of national and international honeys in Saudi Arabia. Arab. J. Chem..

[38] Langner E., Rzeski W. (2014). Biological Properties of Melanoidins: A Review. Int. J. Food Prop..

[39] Tressl R., Wondrak G.T., Garbe L.-A., Krüger R.-P., Rewicki D. (1998). Pentoses and Hexoses as Sources of New Melanoidin-like Maillard Polymers. J. Agric. Food Chem..

[40] Cämmerer B., Kroh L. (1995). Investigation of the influence of reaction conditions on the elementary composition of melanoidins. Food Chem..

[41] Cämmerer B., Jalyschko W., Kroh L.W. (2002). Intact Carbohydrate Structures as Part of the Melanoidin Skeleton. J. Agric. Food Chem..

[42] Hofmann T. (1998). Studies on the Relationship between Molecular Weight and the Color Potency of Fractions Obtained by Thermal Treatment of Glucose/Amino Acid and Glucose/Protein Solutions by Using Ultracentrifugation and Color Dilution Techniques. J. Agric. Food Chem..

[43] Habib H.M., Al Meqbali F.T., Kamal H., Souka U.D., Ibrahim W.H. (2014). Physicochemical and biochemical properties of honeys from arid regions. Food Chem..

[44] Aazza S., Lyoussi B., Antunes D., Miguel M.G. (2014). Physicochemical characterization and antioxidant activity of 17 commercial Moroccan honeys. Int. J. Food Sci. Nutr..

[45] Fernandeztorres R., Perezbernal J., Bellolopez M., Callejonmochon M., Jimenezsanchez J., Guiraumperez A. (2005). Mineral content and botanical origin of Spanish honeys. Talanta.

[46] Al-Mamary M., Al-Meeri A., Al-Habori M. (2002). Antioxidant activities and total phenolics of different types of honey. Nutr. Res..

[47] Hodnick W.F., MllosavljeviĆ E.B., Nelson J.H., Pardini R.S. (1988). Electrochemistry of flavonoids: Relationships between redox potentials, inhibition of mitochondrial respiration, and production of oxygen radicals by flavonoids. Biochem. Pharmacol..

[48] Mayer-Davis E.J., A Bell R., A Reboussin B., Rushing J., A Marshall J., Hamman R.F. (1998). Antioxidant nutrient intake and diabetic retinopathy: The San Luis Valley Diabetes Study. Ophthalmology.

[49] Villanueva V.R., Barbier M., Gonnet M., Lavie P. (1970). The flavonoids of propolis. Isolation of a new bacteriostatic substance: Pinocembrin (dihydroxy-5, 7 flavanone). Ann. L’institut Pasteur.

[50] El-Haskoury R., Kriaa W., Lyoussi B., Makni M. (2018). Ceratonia siliqua honeys from Morocco: Physicochemical properties, mineral contents, and antioxidant activities. J. Food Drug Anal..

[51] Vinson J.A., Hontz B.A. (1995). Phenol Antioxidant Index: Comparative Antioxidant Effectiveness of Red and White Wines. J. Agric. Food Chem..

[52] Alves A., Ramos A., Gonçalves M.M., Bernardo M., Mendes B. (2013). Antioxidant activity, quality parameters and mineral content of Portuguese monofloral honeys. J. Food Compos. Anal..

[53] Estevinho L., Pereira A.P., Moreira L., Dias L.G., Pereira E. (2008). Antioxidant and antimicrobial effects of phenolic compounds extracts of Northeast Portugal honey. Food Chem. Toxicol..

[54] Silici S., Sagdic O., Ekici L. (2010). Total phenolic content, antiradical, antioxidant and antimicrobial activities of Rhododendron honeys. Food Chem..

[55] Silici S., Uluozlu O.D., Tuzen M., Soylak M. (2008). Assessment of trace element levels in Rhododendron honeys of Black Sea Region, Turkey. J. Hazard. Mater..

[56] Pauliuc D., Dranca F., Oroian M. (2020). Antioxidant Activity, Total Phenolic Content, Individual Phenolics and Physicochemical Parameters Suitability for Romanian Honey Authentication. Foods.

[57] Wilczynska A. (2010). Phenolic content and antioxidant activity of different types of polish honey-a short report. Pol. J. Food Nutr. Sci..

[58] Mellen M., Fikselova M., Mendelova A., Hascik P. (2015). Antioxidant Effect of Natural Honeys Affected by Their Source and Origin. Pol. J. Food Nutr. Sci..

[59] Bertoncelj J., Doberšek U., Jamnik M., Golob T. (2007). Evaluation of the phenolic content, antioxidant activity and colour of Slovenian honey. Food Chem..

[60] Becerril-Sánchez A.L., Quintero-Salazar B., Dublán-García O., Escalona-Buendía H.B. (2021). Phenolic Compounds in Honey and Their Relationship with Antioxidant Activity, Botanical Origin, and Color. Antioxidants.

[61] Tuberoso C.I.G., Jerković I., Bifulco E., Marijanović Z. (2011). Biodiversity of *Salix* spp. honeydew and nectar honeys determined by RP-HPLC and evaluation of their antioxidant capacity. Chem. Biodivers..

[62] Dabeek W.M., Marra M.V. (2019). Dietary Quercetin and Kaempferol: Bioavailability and Potential Cardiovascular-Related Bioactivity in Humans. Nutrients.

[63] Guerrero J.A., Lozano M.L., Castillo J., Benavente-García O., Vicente V., Rivera J. (2005). Flavonoids inhibit platelet function through binding to the thromboxane A2 receptor. J. Thromb. Haemost..

[64] Mao W., Schuler M.A., Berenbaum M.R. (2013). Honey constituents up-regulate detoxification and immunity genes in the western honeybee Apis mellifera. Proc. Natl. Acad. Sci. USA.

[65] Socha R., Juszczak L., Pietrzyk S., Fortuna T. (2009). Antioxidant activity and phenolic composition of herbhoneys. Food Chem..

[66] Perna A., Intaglietta I., Simonetti A., Gambacorta E. (2013). A comparative study on phenolic profile, vitamin C content and antioxidant activity of Italian honeys of different botanical origin. Int. J. Food Sci. Technol..

[67] Gašić U., Kečkeš S., Dabić D., Trifković J., Milojković-Opsenica D., Natić M., Tešic Ž. (2014). Phenolic profile and antioxidant activity of Serbian polyfloral honeys. Food Chem..

[68] Santos S.A., Villaverde J.J., Freire C.S., Domingues M.R.M., Neto C.P., Silvestre A.J. (2012). Phenolic composition and antioxidant activity of *Eucalyptus grandis*, *E. urograndis* (*E. grandis* × *E. urophylla*) and *E. maidenii* bark extracts. Ind. Crop. Prod..

[69] Foss K., Przybyłowicz K.E., Sawicki T. (2022). Antioxidant Activity and Profile of Phenolic Compounds in Selected Herbal Plants. Plant Foods Hum. Nutr..

[70] Tounsi S., Wannes W.A., Ouerghemmi I., Jegham S., Njima Y.B., Hamdaoui G., Zemnib H., Moufida B.M. (2011). Juice components and antioxidant capacity of four Tunisian citrus varieties. J. Sci. Food Agric..

[71] Rafa’t Abdul Hassan M.J. (2011). Antimicrobial effect of bee honey on some pathogenic bacteria isolated from infected wounds in comparison to commonly used antibiotics. J. Basrah Res..

[72] Mandal M.D., Mandal S. (2011). Honey: Its medicinal property and antibacterial activity. Asian Pac. J. Trop. Biomed..

[73] Osho A., Bello O. (2010). Antimicrobial effect of honey produced by on some common human pathogens. Asian J. Exp. Biol. Sci..

[74] Taormina P.J., Niemira B.A., Beuchat L.R. (2001). Inhibitory activity of honey against foodborne pathogens as influenced by the presence of hydrogen peroxide and level of antioxidant power. Int. J. Food Microbiol..

[75] Al-Hasani H.M.H. (2018). Study antibacterial activity of honey against some common species of pathogenic bacteria. Iraqi J. Sci..

[76] Hegazi A.G., Al Guthami F.M., Al Gethami A.F.M., Allah F.M.A., Saleh A.A., Fouad E.A. (2017). Potential antibacterial activity of some Saudi Arabia honey. Veter-World.

[77] Ballal M., Shenoy V.P., Shivananda P., Bairy I. (2012). Honey as an antimicrobial agent against Pseudomonas aeruginosa isolated from infected wounds. J. Glob. Infect. Dis..

[78] Molan P.C. (1992). The antibacterial activity of honey: 1. The nature of the antibacterial activity. Bee World.

[79] Dinkov D. (2017). Perspection of Royal Jelly and Bee Honey as new antibacterial therapy agents of hospital infections. J. Clin. Path. Lab. Med..

[80] Irish J., Blair S., Carter D.A. (2011). The Antibacterial Activity of Honey Derived from Australian Flora. PLoS ONE.

[81] López-Hidalgo C., Meijón M., Lamelas L., Valledor L. (2021). The Rainbow Protocol: A Sequential Method for Quantifying Pigments, SUGARS, Free Amino Acids, Phenolics, Flavonoids and MDA from a Small Amount of Sample.

[82] Kadyan S., Rashmi H., Pradhan D., Kumari A., Chaudhari A., Deshwal G.K. (2021). Effect of lactic acid bacteria and yeast fermentation on antimicrobial, antioxidative and metabolomic profile of naturally carbonated probiotic whey drink. LWT.

[83] Dunseath G.J., Bright D., Jones C., Dowrick S., Cheung W., Luzio S.D. (2019). Performance evaluation of a self-administered home oral glucose tolerance test kit in a controlled clinical research setting. Diabet. Med..

[84] Taleb M., Bakour M., Brahim A.T., Ghaber S.M., Abdem S.A.E., Mohamed A., Lyoussi B. (2023). Molecular Characterization of Erythrocyte Glucose-6-Phosphate Dehydrogenase Deficiency in Different Ethnic Groups of Blood Donors in Mauritania. Front. Biosci..

[85] Sunkesula M.S.B., Reddy M.S. (2021). Physico-biochemical and bioactive properties of honey samples from southern india. J. Adv. Sci. Res..

[86] Salim S.N.M., Ramakreshnan L., Fong C.S., Wahab R.A., Rasad M.S.B.A. (2019). In-vitro cytotoxicity of Trigona itama honey against human lung adenocarcinoma epithelial cell line (A549). Eur. J. Integr. Med..

[87] Moniruzzaman M., Khalil M.I., Sulaiman S.A., Gan S.H. (2013). Physicochemical and antioxidant properties of Malaysian honeys produced by *Apis cerana*, *Apis dorsata* and *Apis mellifera*. BMC Complement. Altern. Med..

[88] Mesbahi M.A., Ouahrani M.R., Rebiai A., Amara D.G., Chouikh A. (2019). Characterization of *Zygophyllum album* L. monofloral honey from El-Oued, Algeria. Curr. Nutr. Food Sci..

[89] Marinova G., Batchvarov V. (2011). Evaluation of the methods for determination of the free radical scavenging activity by DPPH. Bulg. J. Agric. Sci..

[90] Lamuela-Raventós R.M. (2018). Folin-Ciocalteu method for the measurement of total phenolic content and antioxidant capacity. Measurement of Antioxidant Activity & Capacity Recent Trends and Applications.

[91] Seal T. (2016). Quantitative HPLC analysis of phenolic acids, flavonoids and ascorbic acid in four different solvent extracts of two wild edible leaves, *Sonchus arvensis* and *Oenanthe linearis* of North-Eastern region in India. J. Appl. Pharm. Sci..

